# ACKR3 expression on diffuse large B cell lymphoma is required for tumor spreading and tissue infiltration

**DOI:** 10.18632/oncotarget.18844

**Published:** 2017-06-29

**Authors:** Viola Puddinu, Sabrina Casella, Egle Radice, Sylvia Thelen, Stefan Dirnhofer, Francesco Bertoni, Marcus Thelen

**Affiliations:** ^1^ Institute for Research in Biomedicine, Università della Svizzera italiana, Bellinzona, Switzerland; ^2^ Graduate School for Cellular and Biomedical Sciences, University of Bern, Bern, Switzerland; ^3^ Institute of Pathology, University Hospital, University of Basel, Basel, Switzerland; ^4^ Institute of Oncology Research, Bellinzona, Switzerland

**Keywords:** ACKR3, CXCR4, chemokine, B cell, lymphoma

## Abstract

Diffuse large B cell lymphoma (DLBCL) is the most frequent lymphoma accounting for more than the 30% of the cases. Involvement of extranodal sites, such as bone marrow and central nervous system, is associated with poor prognosis. A contribution of the chemokine system in these processes is assumed as it is known as a critical regulator of the metastatic process in cancer. The atypical chemokine receptor 3 (ACKR3), which does not couple to G-proteins and does not mediate cell migration, acts as a scavenger for CXCL11 and CXCL12, interfering with the tumor homing CXCL12/CXCR4 axis. Here, functional expression of ACKR3 in DLBCL cells was necessary for colonization of the draining lymph node in an *in vivo* subcutaneous lymphoma model. Moreover, in a disseminated *in vivo* lymphoma model, ACKR3 expression was required for bone marrow and brain invasion and local tumor growth. The present data unveil ACKR3 as potential therapeutic target for the control of tumor dissemination in DLBCL.

## INTRODUCTION

The importance of the chemokine system for orchestrating the homeostatic and inflammatory leucocyte trafficking is amply described. In addition, the chemokine system has critical roles during normal development as well as in pathological tissue growth and aberrant activity of the system was found to have diverse pathogenic consequences, including the onset and the course of diseases [1, 2]. A hallmark of chemokine induced signaling is stimulation of cell migration and requires binding to cognate cell surface chemokine receptors, members of the rhodopsin family of G-protein coupled receptors (GPCRs) [3]. A group of structurally and phylogenetically related chemokine binding proteins was recently classified as atypical chemokine receptors (ACKRs), based on their inability to couple and activate heterotrimeric G-proteins. Presumably due to the lack of G-protein coupling ACKRs are unable to stimulate cell migration [4, 5]. The main function of ACKRs is to scavenge chemokines. The ACKRs are critical players in the resolution of inflammation by removing the excess of chemokines and markedly contribute to gradient formation promoting efficient leukocyte trafficking [6–8].

ACKR3 is a scavenger of CXCL11 and CXCL12, which are ligands of the typical receptors CXCR3 and CXCR4, respectively. Noteworthy, ACKR3 has approximately 10 fold higher affinity for CXCL12 than CXCR4 (IC_50_=0.4 nM vs. 3.6 nM) [9–11]. During B cell development ACKR3 is upregulated at the plasmablast stage and due to its scavenging activity was suggested to license these cells to leave the CXCL12-rich environment of germinal centers (GC) in B cell follicles of secondary lymphoid organs [12]. On the other hand, local scavenging of CXCL12 by ACKR3 markedly contributes to the formation of functional chemotactic gradients [7, 13, 14].

CXCR4 and ACKR3 as well as CXCL12 are frequently overexpressed in human cancers where they contribute to tumor growth and metastasis formation [1, 15–18]. Elevated expression of CXCR4 is frequently associated with unfavorable prognosis [19]. Similarly, ACKR3 was found to be upregulated in many solid tumors including breast, lung, prostate and hepatocellular cancers, neuroblastoma, glioma, and cutaneous squamous cell carcinoma [20–27].

Nevertheless, the molecular mechanism of ACKR3 in tumor formation is less clear and may vary between cancers types. In some tumors ACKR3 was shown to affect CXCL12/CXCR4 signaling [18, 28, 29]. ACKR3 can also indirectly regulate CXCR4 surface expression by modulating CXCL12 levels. In breast cancer, ACKR3 expressed *in trans* can modulate CXCL12 levels leading to altered CXCR4-dependent tumor growth [24]. In the absence of ACKR3, CXCL12 can accumulate and lead to the downregulation and degradation of CXCR4 [30, 31]. ACKR3 can also influence tumor vascularization by regulating CXCL12 levels [32]. The described controversial roles of ACKR3 in tumor formation and metastasis do not allow making general predictions.

Few studies address the role of ACKR3 in hematological cancers. The receptor is markedly upregulated in acute lymphoblastic leukemia (ALL) [33] and acute myeloid leukemia (AML) [34]. In mucosa-associated lymphoid tissue (MALT) neoplasms upregulation of ACKR3 and concomitant downregulation of CXCR4 could play a role in the transformation to diffuse large B-cell lymphoma (DLBCL) [35, 36]. Typically, DLBCL arise from GC cells, either from centroblast leading to GC B-cell like (GCB), or from plasmablasts leading to activated B cell-type (ACB) lymphomas [37]. DLBCL is the most frequent lymphoma and accounts for about 30% of all newly diagnosed cases and frequently involves extranodal sites [37]. Invasion of bone marrow occurs in 10-15% of patients [38], whereas involvement of the central nervous system (CNS) occurs in about 5% of cases and is associated with very poor prognosis [39]. Here we investigated the role of ACKR3 on the DLBCL cell line VAL. In a xenograft model in immunodeficient mice cell surface expression of functional active ACKR3 becomes markedly upregulated without alterations of its mRNA expression. Genetic ablation of ACKR3 by CRISPR/Cas9 attenuates cell migration *in vivo* and markedly limits tissues invasion of the lymphoma cells.

## RESULTS

### Subcutaneous conditioning increases surface expression of ACKR3

**Figure 1:**
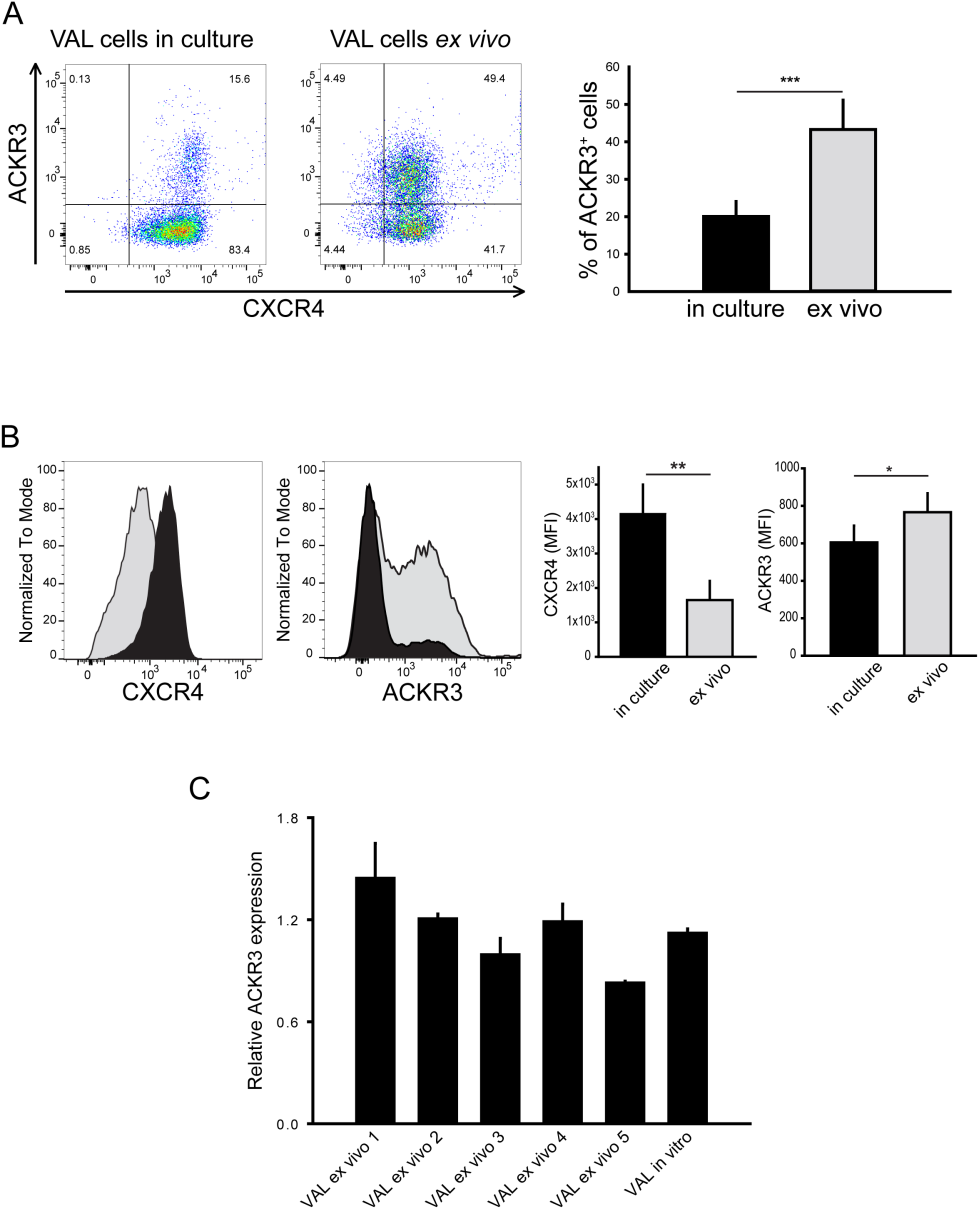
ACKR3 surface expression is upregulated on *ex vivo* cells without affecting ACKR3 gene transcription levels **(A)** Surface expression of ACKR3 and CXCR4 on VAL cells in culture or extracted from localized xenografts (*ex vivo*) grown in NOD/SCID/common γ-chain^ko^ mice. For FACS analysis cells were stained with the anti-human ACKR3 mAb 9C4 and the anti-human CXCR4 mAb 12G5. Representative data are shown for one of six xenografts from three independent experiments (n=5-16 mice per experiment). Quantification of the percentage of ACKR3-expressing cells reported as mean ±SD. **(B)** Data as in A presented as histograms to reveal differences in relative receptor surface expression. Left, gray histograms VAL cells *ex vivo*, black histograms VAL cells in culture. Right, quantification of the mean fluorescence intensities (MFI) reported as mean ±SD. Data from three independent experiments. Statistical analysis (*** = p<0.0001; ** = *p*<0.001; * = p<0.05) was performed with two-tailed Student’s t-test. **(C)** Relative ACKR3 transcription levels of human CD19^+^-enriched VAL cells extracted from five different xenografts (VAL *ex vivo* 1 to 5) and VAL cells in culture assessed by RT-PCR. Results were normalized against human TBP1 mRNA levels and are expressed as 2^-ΔCt^. Histograms report mean ACKR3 expression ± SEM measured as triplicates. Representative plot from one of two independent experiments.

The observation that ACKR3 is upregulated in human plasmablasts, prompted us to interrogate the expression of its mRNA in human DLBCL lines. The transcript of ACKR3 was found in several, but not all DLBCL lines tested. By semi quantitative PCR analysis VAL cells showed a moderate, but consistent expression of ACKR3 and were therefore selected for the subsequent experiments (Supplementary Figure 1A). Despite being clearly expressed at the mRNA level, only about 15% of VAL cells expressed ACKR3 on the cell surface. FACS analysis using different monoclonal antibodies, i.e. clones 9C4 [11] (Figure 1A) and clone 11G8 [10] (Supplementary Figure 1B), revealed the presence of two populations with and without ACKR3 present on the plasma membrane. By contrast, all VAL cells expressed similar levels of CXCR4 on the cell surface, which renders them a suitable model for studying ACKR3 modulation of the CXCR4/CXCL12 axis. When VAL cells were sorted for ACKR3 surface expression both populations, ACKR3^+^ and ACKR3^-^, showed similar levels of mRNA transcripts (Supplementary Figure 1B). The finding suggests that in VAL cells ACKR3 may preferentially localize in intracellular compartments as reported for other leukocytes [33, 34, 40]. Both, ACKR3 positive and negative sorted cells reverted to the same phenotype of unsorted cells after 2-3 weeks of culture indicating a dynamic equilibrium of the populations (data not shown). Tumor environment is often characterized by reduced oxygen supply. *In vitro*, the hypoxia-mimetics cobalt chloride and DMOG [41] efficiently upregulate CXCR4, but not ACKR3, on VAL cells (Supplementary Figure 2), indicating that compartmentalization of the typical and atypical chemokine receptors is controlled by different mechanisms and is intrinsic to the cells.

The aggressiveness of DLBCL cell lines RIVA and TOLEDO, when injected into NOD/SCID immunosuppressed mice, positively correlated with CXCR4 surface expression. Conditioning of RIVA cells in subcutaneous localized tumors further triggered tissue invasiveness and lethality, when such cells were injected intravenously [42]. However, compared to RIVA cells, VAL cells expressed higher levels of ACKR3, but similar levels of CXCR4 mRNA (not shown) and did not upregulate CXCR4 surface expression when grown in subcutaneous xenografts in NOD/SCID/common γ-chain^ko^ mice (Figure 1A and 1B). Moreover, Figure 1A and 1B reveals that all Val cells expressed similar percentages of CXCR4^+^ cells; however, the *in vivo* passage moderately decreased the surface expression level of CXCR4 on the ACKR3^+^ VAL cells (Figure 1B). By contrast, ACKR3 became markedly upregulated as up to 50% of the conditioned cells (*ex vivo*) expressed the receptor (Figure 1A and 1B), indicating that after localized tumor passage the presence of two populations existed, namely ACKR3^+^/CXCR4^+^ and ACKR3^-^/CXCR4^+^cells. After 2-3 weeks of *in vitro* culture, *ex vivo* cells were phenotypically indistinguishable from the starting cells, indicating that the environment influences ACKR3 surface expression. More importantly, the high surface ACKR3 expression of *ex vivo* cells isolated from localized tumors, was not accompanied by variations of *ACKR3* gene transcripts measured by RT-PCR (Figure 1C) mirroring the expression of ACKR3^+^ sorted cells.

### ACKR3 is functional on *ex vivo* VAL cells

**Figure 2:**
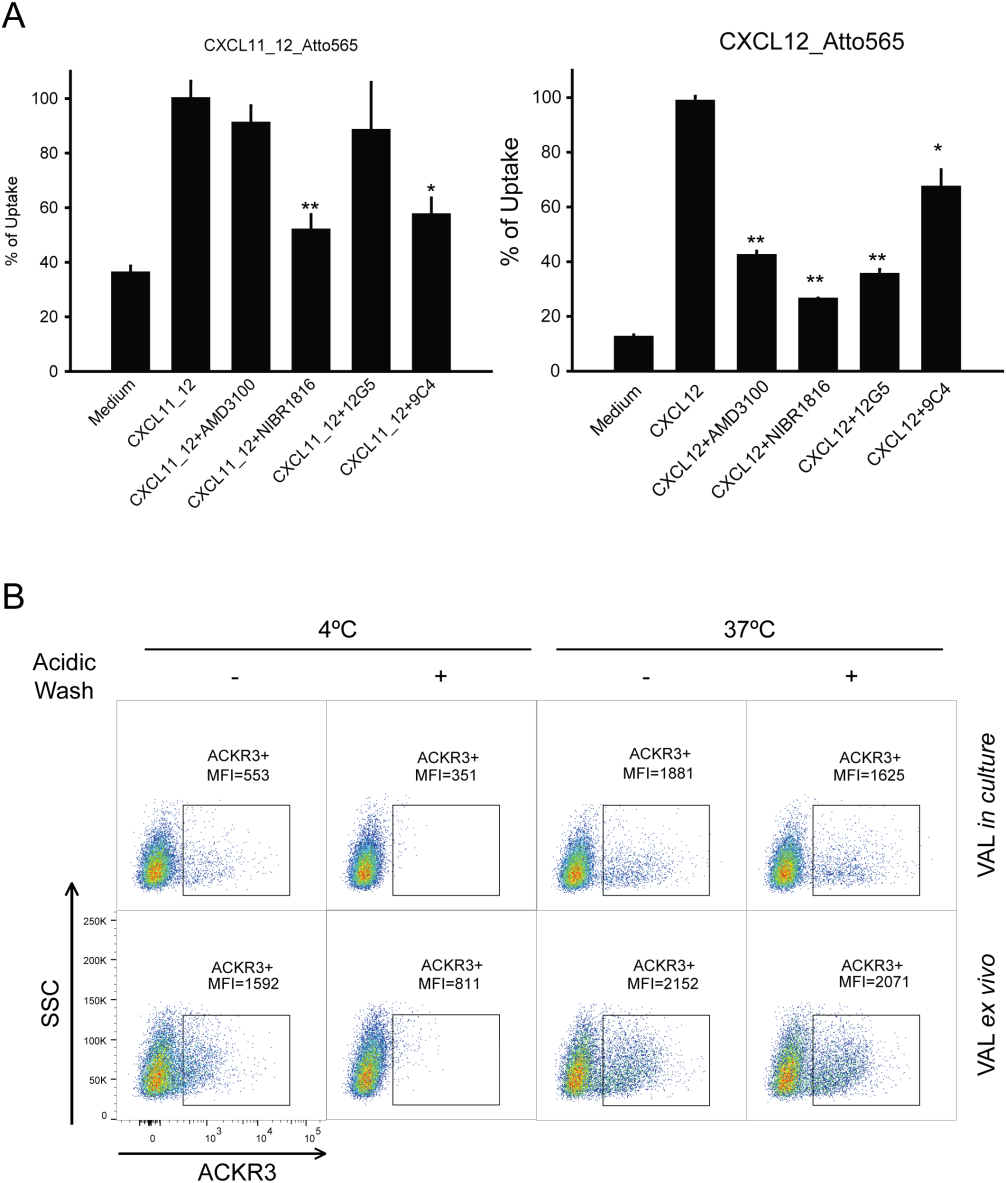
ACKR3 is functional on VAL cells **(A)** ACKR3-dependent uptake of the fluorescent-labelled chimeric chemokine CXCL11_12 (left panel) and CXCL12 (right panel) (50 nM each, 1 h at 37°C) was analyzed by flow cytometry on *ex vivo* VAL cells. Cells were preincubated for 30 min with the ACKR3-blocking mAb 9C4 (30 μg/ml), the CXCR4 inhibitor NIBR1816 (5 μM), the CXCR4 inhibitor AMD3100 (10 μM) or the anti-CXCR4 mAb 12G5 (30 μg/ml). Plots report mean percentages of Atto565 MFI ±SD of maximum uptake observed with 50 nM chemokine alone. Statistical analysis was performed with ANOVA one-way test *p<0.032, **p<0.019. Representative results of *ex vivo* VAL cells from two independent experiments performed in duplicates. **(B)** Surface binding and uptake of anti-ACKR3 mAb 11G8-PE by *ex vivo* and in culture VAL cells. Cells were incubated at 4°C 15 min and then incubation was continued for 1h at 4°C or 37°C with the mAb. When indicated, surface bound 11G8-PE was removed with a brief acidic wash at pH=3. Samples analyzed by flow cytometry, one of three independent experiments is shown.

Different functional assays were used to test ACKR3 activity on VAL cells. Previously we have shown that ACKR3-mediated uptake of ligands is a reliable method to determine its activity on B cells [43]. In order to assess ACKR3-mediated chemokine binding and internalization in the presence of CXCR4, an ACKR3-specific chimeric chemokine (CXCL11_12), consisting of CXCL12 body with the N-terminal sequence of CXCL11, the second ligand of ACKR3 was generated, in line with the notion that modifications of the N-terminus abrogate binding of CXCL12 to CXCR4 [44]. In fact, CXCL11_12 selectively binds ACKR3, but not CXCR4 and CXCR3 (MT and ER, unpublished). *Ex vivo* VAL cells incubated with 50 nM of CXCL11_12 labeled with Atto565 [45] efficiently internalized the chimeric chemokine (Figure 2A). Uptake was markedly attenuated by the mAb 9C4, but not by the small molecule AMD3100 and the mAb 12G5, which both target CXCR4, indicating that CXCL11_12 was internalized by the scavenger ACKR3. Interestingly, incubation with the CXCR4 inhibitor isothiourea NIBR1816 [46] significantly affected CXCL11_12 uptake. NIBR1816 was shown to insert into the binding cavity of CXCR4 and to interact with selective side chains [47], that are considered critical for receptor activation. These residues (e.g. Asp97 and Asp187) are not conserved in ACKR3, suggesting a different binding mechanism to the scavenger, that interferes with CXCL11_12 uptake. When the cells were incubated with CXCL12 instead, the uptake became markedly sensitive to AMD3100 and the mAb 12G5, but remained sensitive to mAb 9C4 indicating that internalization was mediated by both, CXCR4 and ACKR3. In agreement with the internalization of CXCL11_12, the strongest inhibition of CXCL12 uptake was observed in the presence of NIBR1816 in line with the conclusion that the compound targets both receptors (Figure 2A).

Next, binding and uptake of an ACKR3-specific antibody was investigated. For antibody binding to surface expressed receptors VAL cells were kept at 4°C, whereas incubation at 37°C allowed receptor internalization. In accordance with the higher surface expression of ACKR3, *ex vivo* VAL cells internalized more efficiently fluorophore-conjugated ACKR3-specific mAb 11G8 than cells kept in culture (Figure 2B). Internalization was confirmed by the resistance of antibody-derived fluorescence to a brief acidic wash which removes surface bound mAb [48]. Moreover, at 4°C when endocytic processes are blocked, more antibody bound to *ex vivo* cells compared to cells kept in culture recapitulating the higher ACKR3 surface expression (Figure 2B).

### Creation of VAL ACKR3^ko^ cells with the CRISPR/Cas9 system

**Figure 3:**
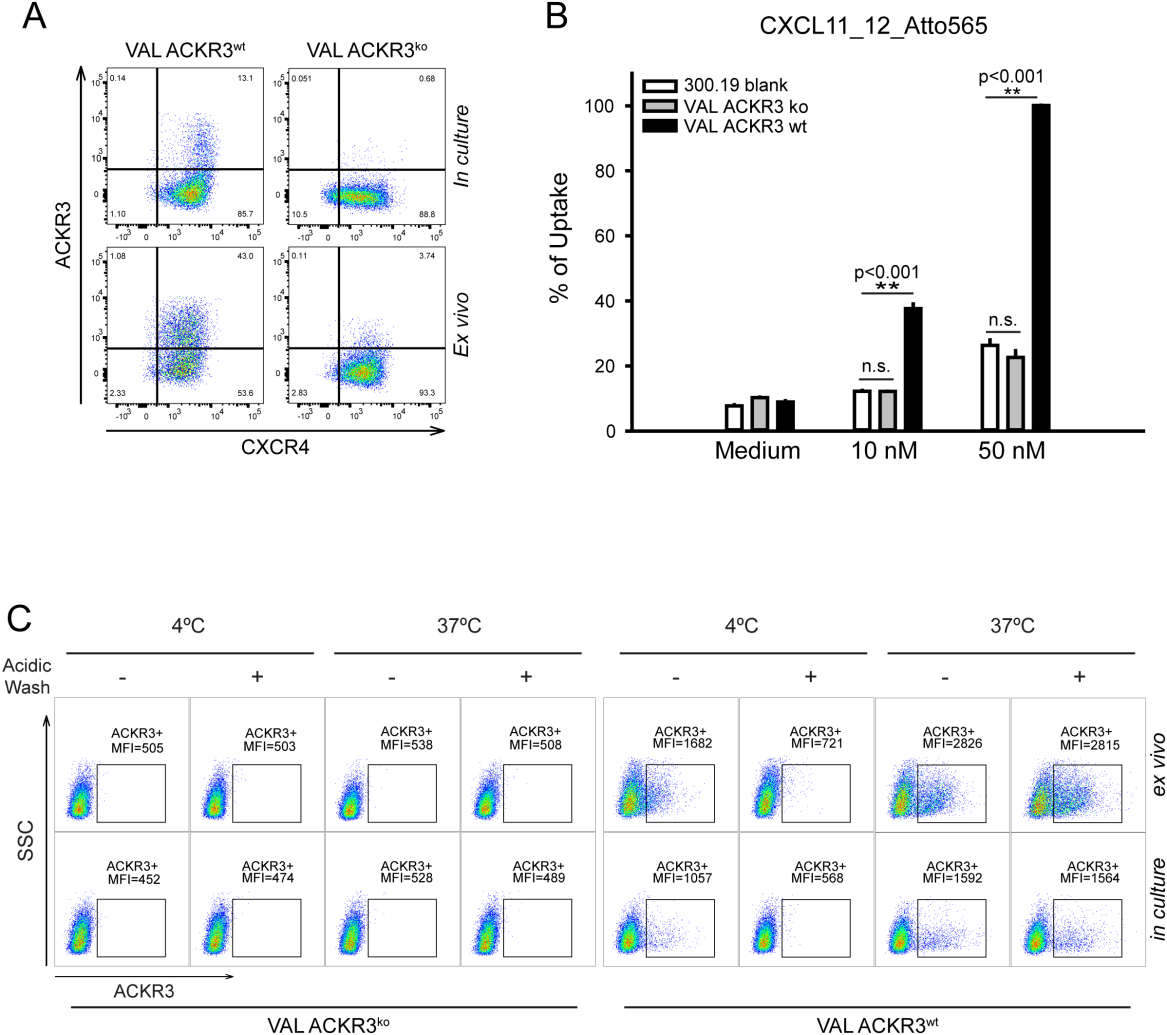

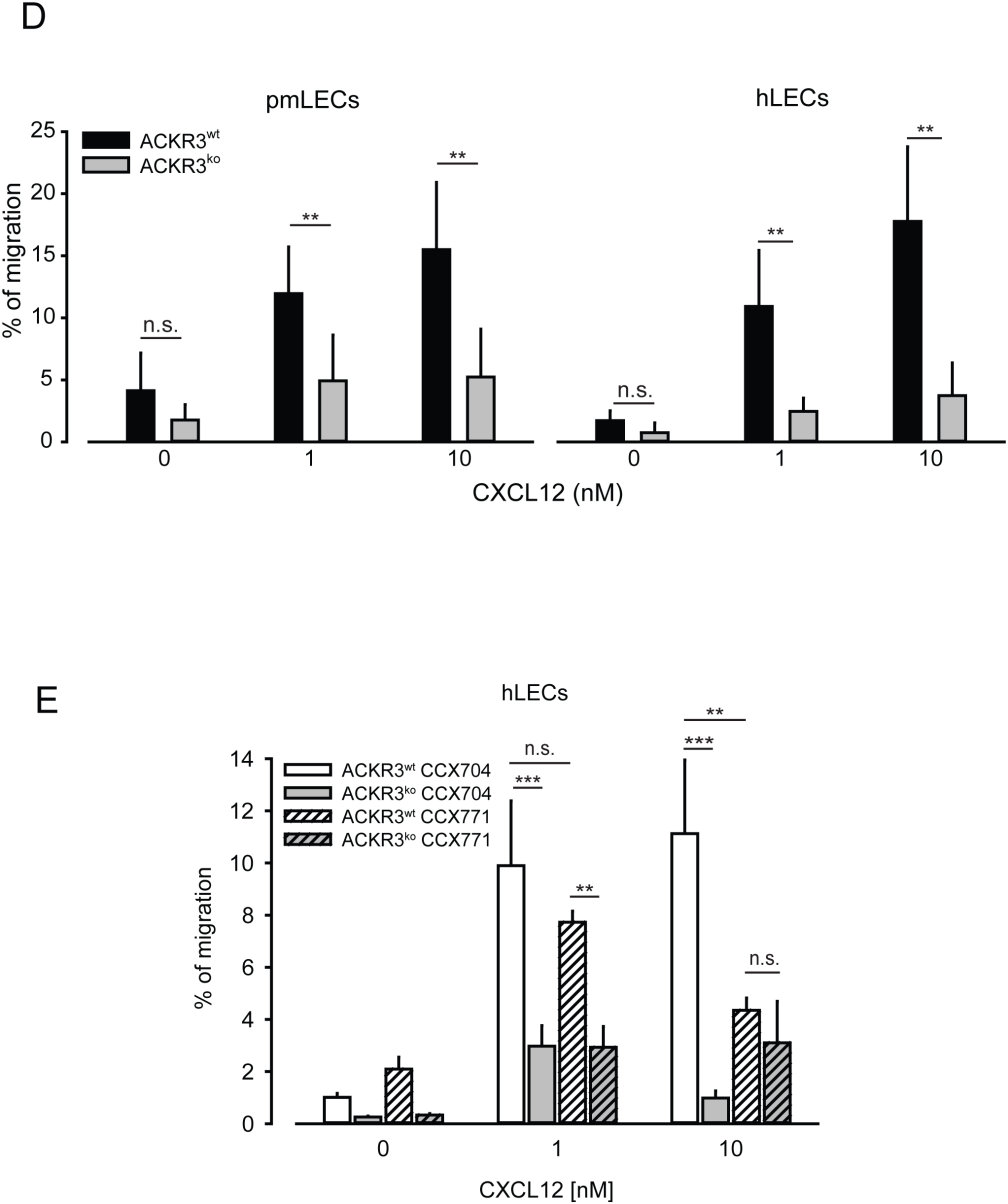
Characterization of ACKR3^ko^ VAL cells **(A)** ACKR3 (mAb 9C4) and CXCR4 (mAb 12G5) surface expression on VAL cells grown in culture or extracted from localized tumors (*ex vivo*). One out of over 10 observations. **(B)** ACKR3^wt^and ACKR3^ko^ VAL cells and the ACKR3 deficient pre-B cell line 300.19 (blank) were incubated at 37°C for 1 hour with the ACKR3-specific chimeric chemokine CXCL11_12 by. Uptake was measured by FACS as percentage of maximum uptake observed with 50 nM CXCL11_12, **p<0.001, n.s.= not significant, statistical analysis was performed with one-way ANOVA test. Representative plot from duplicates of three independent experiments. **(C)** Surface binding and uptake of mAb 11G8-PE by ACKR3^wt^ and ACKR3^ko^VAL cells kept in culture or extracted from localized tumors as in figure 2. Results of one of three independent experiments are shown (10 mice per group). **(D)** Transendothelial migration of *ex vivo* VAL cells. VAL cell were allowed to migrate for 6h through pmLEC, primary mouse lymphatic endothelium and hLECs, human lymphatic endothelium. **(E)** *Ex vivo* ACKR3^wt^and ACKR3^ko^ VAL cells were allowed to migrate for 6h through hLECs in the presence of 1μM CCX704 (inactive control) and CCX771. **(D, E)** Statistical analysis was performed with two-way ANOVA test. **p<0.01, ***p<0.001, n.s.= not significant.

To test the role of ACKR3 in tumor growth of VAL cells, the genome editing CRISPR/Cas9 technology was used to eliminate the receptor [49]. With this method the ACKR3 gene was disrupted and cyan fluorescent protein (CFP) inserted. Genomic PCR analysis of a CFP positive clone revealed the complete modification of the *ACKR3* gene locus of all alleles (Supplementary Figure 3). Figure 3A shows the FACS analysis of VAL ACKR3^wt^ and ACKR3^ko^ cells, which were kept in culture or conditioned in a localized subcutaneous xenograft. Typical staining of ACKR3 was observed in wild type VAL cells (Figure 1A), which was completely abolished in ACKR3^ko^ cells and was also absent in corresponding *ex vivo* cells. Cells lacking *ACKR3* gene completely lost the ability to uptake CXCL11_12. As control mouse 300.19 pre-B cells which lack ACKR3 expression were used as controls for residual non-specific binding (Figure 3B). Similar, binding and uptake of the ACKR3-specific mAb 11G8 was abolished in ACKR3^ko^ VAL cells either in culture or *ex vivo* (Figure 3C).

The chemotactic activity of ACKR3^wt^ and ACKR3^ko^ VAL cells in response to CXCL12 was tested by *in vitro* transmigration through human and mouse lymphatic endothelium (Figure 3D). Surprisingly, ACKR3 deletion in VAL cells markedly attenuated their chemotactic activity. However, when applied to ACKR3^wt^ cells, indeed CCX771, but not the inactive compound CCX704, attenuated transendothelial migration. An inhibitory effect of CCX771 on the migration of ACKR3^ko^ cells could not be detected due to their marginal response to CXCL12 (Figure 3E). The observation contrasts previous reports were it was shown that ACKR3 agonists such as CXCL11 or the small compound CCX771, which antagonize CXCL12 binding, but stimulate β-arrestin2 recruitment, attenuate transendothelial migration (TEM) [50]. Noteworthy, in ACKR3^ko^ VAL cells the receptor was absent and therefore unable to interact with arrestin.

### Ablation of ACKR3 function reduces tumor cell infiltration

**Figure 4:**
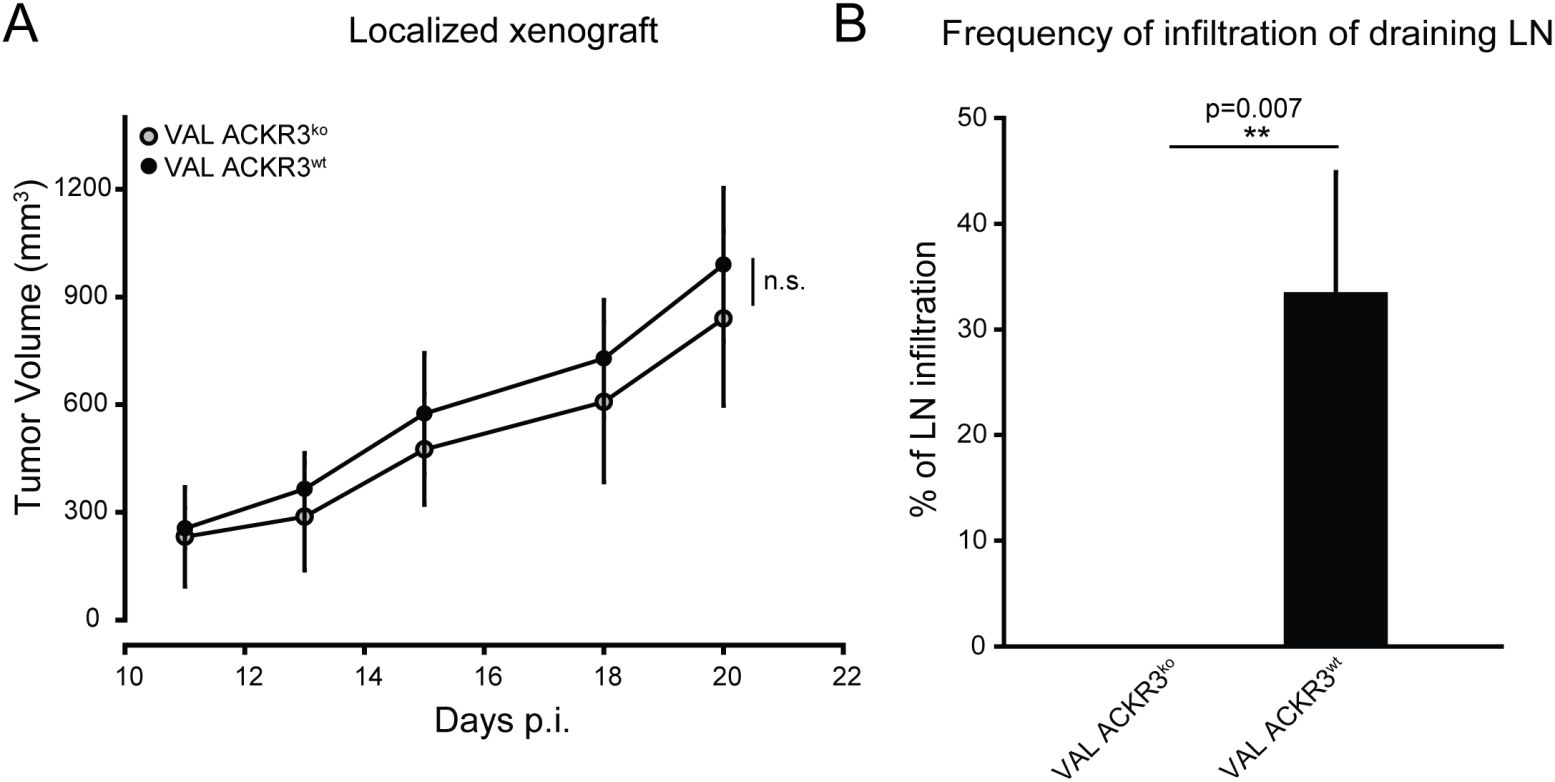
ACKR3 ablation does not affect local tumor growth but attenuates spreading to draining lymph nodes **(A)** NOD/SCID/common γ-chain^ko^ mice (n=10) were injected subcutaneously with 10^7^ VAL ACKR3^ko^ and VAL ACKR3^wt^ cells. Tumor volume was measured over time. Representative plot of one of three independent experiments. **(B)** Frequency of draining lymph nodes infiltrated with human CD19^+^ tumor cells. Cumulative results of three independent experiments. Statistical analysis was performed with two-tailed Student’s t-test. *p<0.1,**p<0.01, n.s.= not significant.

A localized xenograft model was used to study the role of ACKR3 in VAL DLBCL tumor spreading and tissue infiltration. Subcutaneous injection of 10^7^ ACKR3^wt^ and ACKR3^ko^ VAL cells into flanks of NOD/SCID/common γ-chain^ko^ mice revealed no difference in local tumor growth (Figure 4A). Tumors became measurable with both cell types after 10 days and were followed for about three weeks. In general, cancer cells emigrate from local tumors via lymphatics and first signals of metastasis are found in the draining lymph nodes. Importantly, mice injected with ACKR3^ko^ cells showed markedly lower frequency of infiltration of human CD19^+^ VAL cells into draining lymph nodes compared to animals injected with ACKR3^wt^ VAL cells (Figure 4B). The observation suggests that tumor evasion and spreading of DLBCL VAL cells required ACKR3 expression. Previous data showed that systemic inhibition of CXCL12 scavenging by ACKR3 with small molecule antagonists, such as CCX754 and CCX771, caused markedly increased CXCL12 serum levels [24, 51, 52]. In the present study ACKR3 was genetically deleted on the tumor cells while the receptors expressed on mouse tissue were not inhibited, suggesting that expression of ACKR3 on VAL cells was required for spreading via lymphatics but was independent of elevated CXCL12 levels. Collectively the reduced TEM observed *in vivo* and *in vitro* in the absence of ACKR3 indicates a critical role for the receptor in controlling CXCR4-mediated cell migration.

### Ablation of ACKR3 limits organ infiltration in a disseminated xenograft model

**Figure 5:**
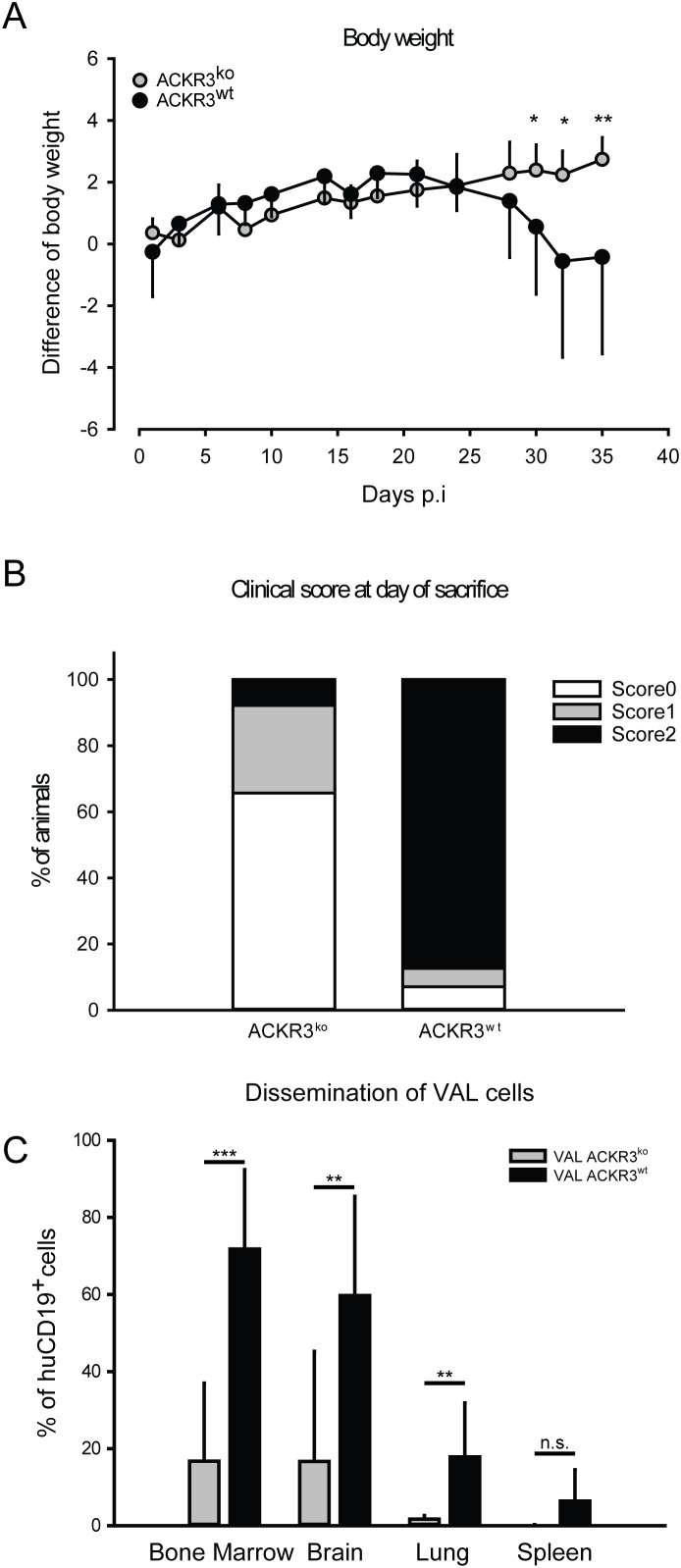
Mice injected with VAL ACKR3^ko^ cells show reduced loss of body weight, milder clinical manifestation and reduced organ invasion in a model of disseminated xenografts **(A)** NOD/SCID/common γ-chain^ko^ mice were intravenously injected with 2×10^5^ cells. Body weight variation over time. **(B)** Clinical scores of ACKR3^ko^and ACKR3^wt^ VAL cells disseminated xenografts. Cumulative results of three independent experiments. **(C)** Percentages of human VAL cells in total extracted cells. Human CD19^+^ were measured by flow cytometry. Three independent experiments with 9 or 10 mice per group, plots report mean ± SD of one representative experiment. p-value was calculated with two-tailed Student’s t-test. ***p<0.0001, **p<0.001, * p< 0.05, n.s.= not significant.

**Figure 6:**
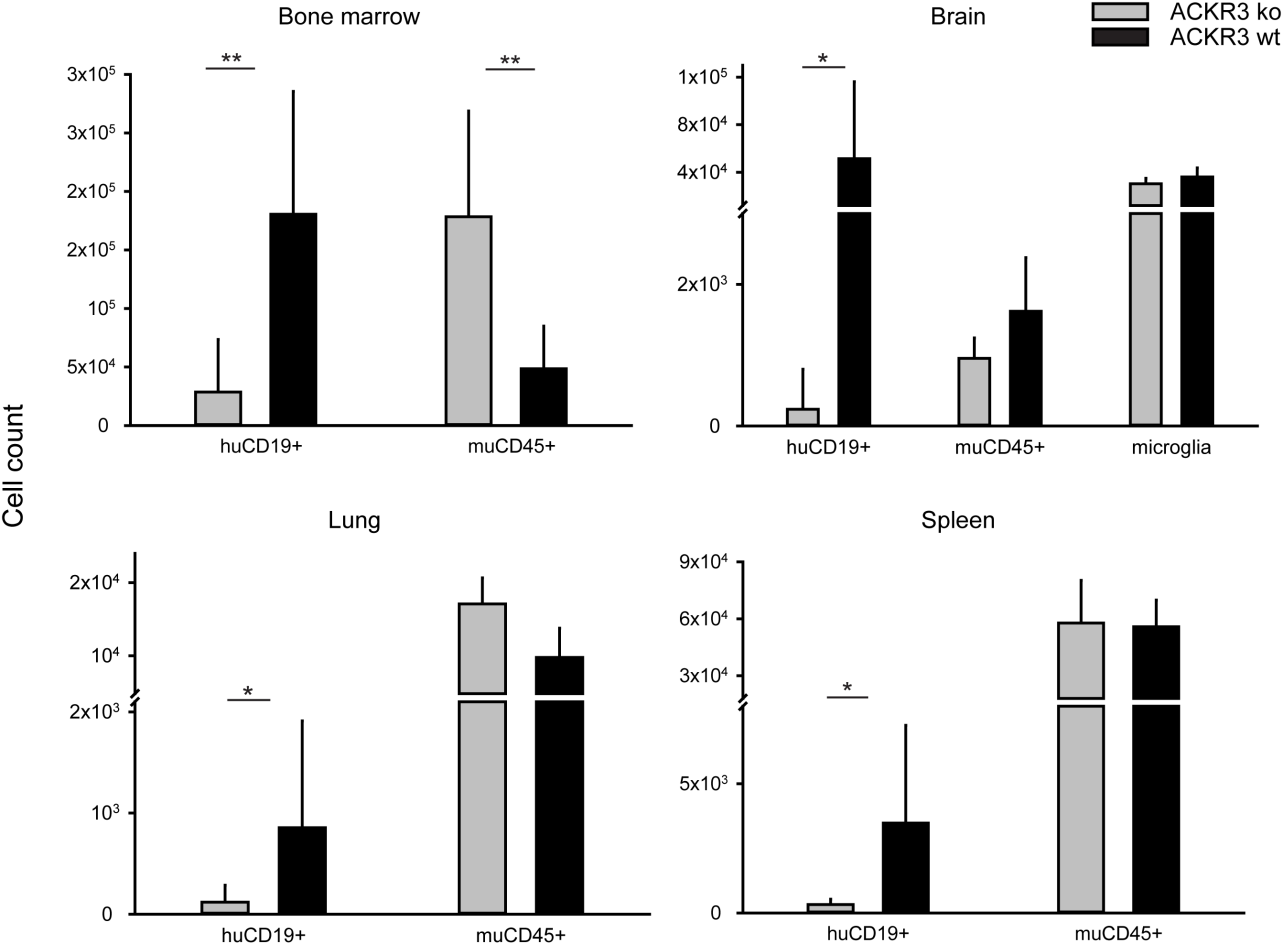
Tissue invasion of VAL ACKR3^ko^ and VAL ACKR3^wt^ cells NOD/SCID/common γ-chain^ko^ mice were injected with 2×10^5^ cells. Organs (bone marrow, brain, lung and spleen) were collected after four weeks and human CD19^+^ cells as well as mouse CD45^+^ leukocytes and in the brain mouse CD45^low^ microglia counted by FACS in the extracts. Cumulative data from one out of three independent experiments with 10 animals per group. Statistical analysis was performed with Student’s t test. * p<0.01, ** p<0.001.

It is well known that chemokine receptors can orchestrate organ-specific metastasis [53]. Disseminated xenograft models, in which cells are directly injected into the circulation, can be used to study the ability of neoplastic cells to colonize organs. In order to test the role of ACKR3 on the capability of VAL cells to infiltrate distant organs 2×10^5^ ACKR3^wt^ and ACKR3^ko^ cells were injected intravenously into NOD/SCID/common γ-chain^ko^ mice. The tumor tissue infiltration was monitored daily for signs of neurologic disorders and when first symptoms of hind-leg paralysis (score 2) manifested all animals were sacrificed [54]. Mice injected with ACKR3^wt^ and ACKR3^ko^ VAL cells initially gained weight at a similar rate, but after about four weeks animals injected with wild type cells markedly lost weight (Figure 5A) which correlated with a remarkably higher clinical score at day of sacrifice (Figure 5B). Organs typical for CXCL12/CXCR4-mediated tumor infiltration, such as bone marrow, brain, lungs and spleens were collected [53]. Organs were processed and stained for human CD19 as a marker for VAL cells and subjected to FACS analysis. Figure 5C shows that ACKR3^wt^ VAL cells displayed a prominent invasion of multiple organs, which was most evident in bone marrow, followed by brain, lung and spleen. By contrast, all organs were markedly less infiltrated by the ACKR3^ko^ VAL cells in line with the lower TEM capability. The cells extracted from the organs were also stained for mouse CD45 as a measure for host leukocytes in the tissues. In bone marrow the frequency of human CD19^+^ VAL inversely correlated with mouse CD45^+^ leukocytes depending on the expression of ACKR3 (Figure 6). The observation suggests that human ACKR3^wt^ VAL cells efficiently compete for mouse CD45^+^ leukocyte niches in bone marrow. The competition was not observed in brain, lung and spleen (Figure 6). Neither was the frequency of mouse microglia cells altered in the brain.

**Figure 7:**
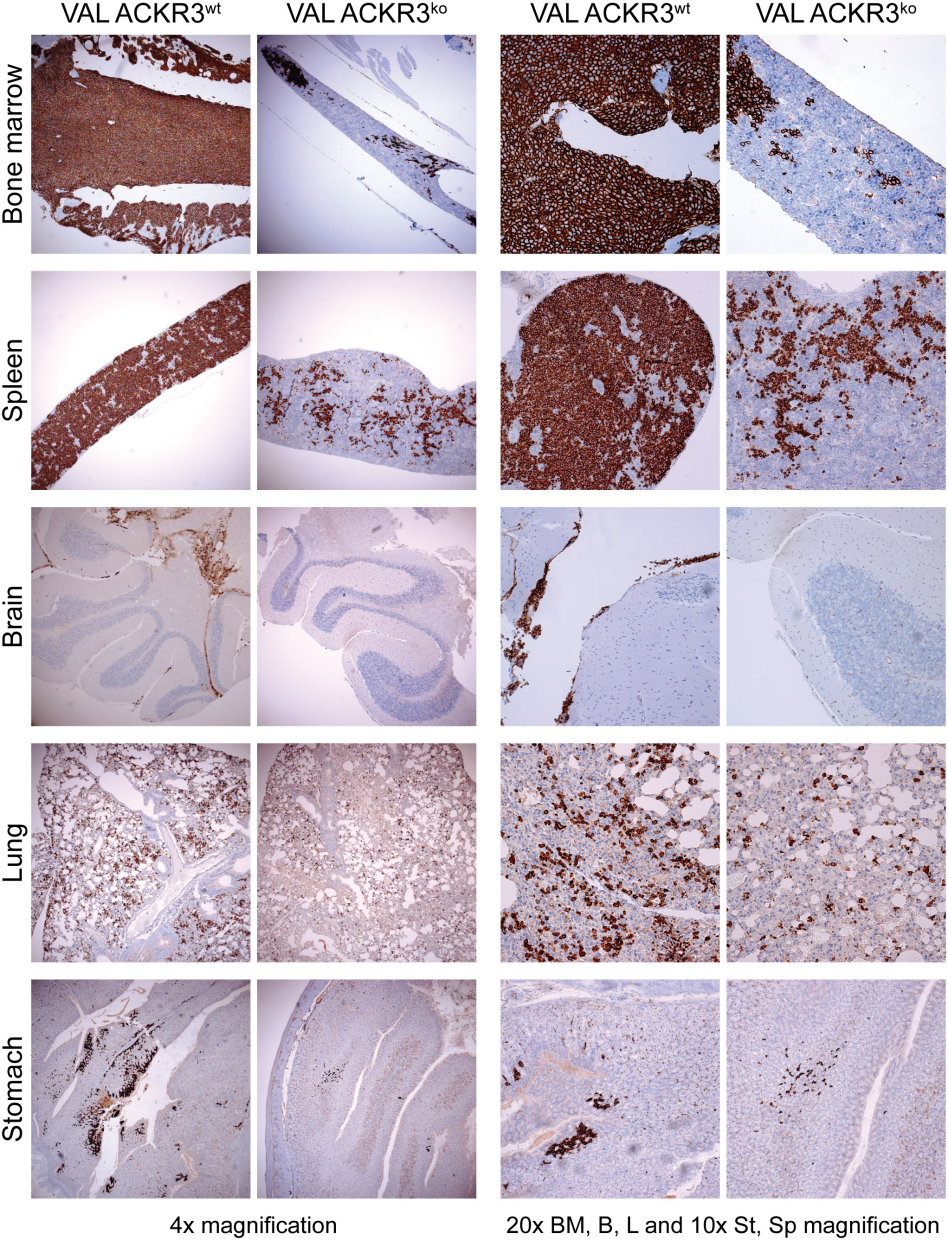
Immunohistochemistry of tissue invasion by VAL ACKR3^KO^ and VAL ACKR3^wt^ cells NOD/SCID/common γ-chain^ko^ mice were injected with 2×10^5^ cells. Organs were removed after four weeks, formalin-fixed, paraffin-embedded and sections stained for CD20 (brown) expressing human VAL cells and counterstained with hematoxylin (blue).

In line with the FACs analyses (Figure 6), immunohistochemistry of paraffin embedded organs from mice injected with ACKR3^wt^ VAL cells showed a higher degree of tissue infiltration compared to animals exposed to ACKR3^ko^ VAL cells (Figure 7). The difference was most prominent in lymphoid organs. ACKR3^wt^ VAL cells diffusely infiltrated the bone marrow almost completely replacing host hematopoiesis, whereas ACKR3^ko^ VAL cells showed only local foci of invasion. Similarly, the spleen from mice injected with ACKR3 wild type cells displayed a widespread infiltration of CD20 positive lymphoma cells, in contrast to the spleen treated with ACKR3 deficient cells which displayed only a focal infiltration. In NOD/SCID/common γ-chain^ko^, which lack mature lymphocytes, lymph nodes are very small if at all detectable. However, in the systemic xenograft model of mice injected with ACKR3^+^ VAL cells, a few enlarged lymph nodes could be found, which were diffusely infiltrated with human CD20^+^ large lymphoma cells (Supplementary Figure 4). In the brain the difference in ACKR3-dependent infiltration of VAL cells was striking. While ACKR3^ko^ VAL cells were almost absent in the brain, ACKR3^wt^ VAL cells showed a predominant meningeal infiltration pattern, with foci of beginning parenchymal invasion at the day of sacrifice (Figure 7, 4x magnification).

In lung, both ACKR3^wt^ and ACKR3^ko^ cells were present, however ACKR3^wt^ cells were more frequent (Figure 7). In the gastrointestinal tract only the stomach displayed some infiltration of VAL cells, which appeared slightly more prominent, when cells express ACKR3. By contrast, the lymphoma cells essentially spared the small intestine and colon (Supplementary Figure 4). Similarly, only a marginal infiltration of the skin was observed (Supplementary Figure 4).

## DISCUSSION

A continuously growing number of publications report elevated ACKR3 expression in many human cancers, including solid tumors and hematological malignancies [18, 28, 29, 33–35, 55]. Various mechanism have been proposed by which ACKR3 may sustain tumor development including promoting tumor growth, dampening apoptosis or favoring metastasis formation [20, 21, 23–27, 56]. Next to its scavenging activity, it is plausible that ACKR3-mediated signaling is responsible for the pathogenic role of the receptor. In line with this, it was recently proposed that stimulation of ACKR3 with CXCL12 leads to the activation of mTOR and Rho/ROCK pathways promoting cell migration and liver metastasis of pancreatic cancer cells [20].

Increased ACKR3 surface expression after *in vivo* passage or sorting of cells is not accompanied by elevated mRNA levels, suggesting a shift in receptor localization from endosomal structures to the cell surface. In line with this, preferential intracellular localization of ACKR3 has been reported [13, 40, 57–59]. However, an increase in ACKR3 protein levels is also possible. Nevertheless, several available mAb did not yield conclusive intracellular staining of ACKR3, in particular when ACKR3^wt^ and ACKR3^ko^ cells where compared. In addition, western blot analysis of membranes from ACKR3^wt^ and ACKR3^ko^ VAL cells were not decisive. Increased surface expression of ACKR3 was accompanied with enhanced scavenging activity (Figure 2B) demonstrating the functionality of the surface expressed receptor. Ligand induced and spontaneous internalization of ACKR3 requires an intact C-terminus [9, 58, 59] and arrestin binding [50, 60, 61]. ACKR3 does not bind G-proteins and was proposed to mediate arrestin-dependent signaling [62]. Moreover, the competitive agonist CCX771 stimulates arrestin recruitment at ACKR3, but efficiently blocked CXCL12/CXCR4-mediated TEM [50]. The inhibitory effect on TEM was attributed to both, the expression of ACKR3 on endothelial cells as well as on the migrating tumor cells [50]. However, a possible ACKR3-mediated cell-cell interaction or agonist-induced modulation of ACKR3 signaling through arrestin were not excluded [50, 60].

The observation that ACKR3^ko^ VAL cells showed markedly reduced CXCL12-stimulated TEM supports the assumption, that ACKR3 mediates a positive signal for CXCR4-mediated TEM through interaction with the endothelium. The fact that deletion of ACKR3 dampens CXCR4-mediated TEM (Figure 3D), argues against an inhibitory arrestin-dependent signaling by ACKR3. On the other hand, because CCX771 has agonistic effects on ACKR3 and enhances arrestin binding [50], it is possible that sequestration of arrestin for binding to CXCR4 leads to potential inhibition of TEM [60]. In line with this, arrestin binding to CXCR4 was reported to be required for CXCL12 dependent cell migration [63–65]. Again, the attenuated TEM of ACKR3^ko^ VAL cells strongly argues against a direct ACKR3-arrestin mediated effect. A potential mechanism of ACKR3-mediated modulation of CXCR4 signaling could rely in allosteric regulation through multimerization, which may not require stable physical interactions, as often proposed for GPCR dimers, since both receptors can internalize independently [66–68]. In fact, it was shown, that ACKR3 and CXCR4 co-expression can enhance CXCL12-stimulated migration in an arrestin dependent manner [68].

Malignant cells often upregulate chemokine receptors allowing tissue specific homing of circulating tumor cells of solid cancers [1, 53] and of several lymphoma subtypes [69]. The typical chemokine receptor CXCR4 is present in many cancers and is responsible for metastasis formation in several organs [1]. The concomitant expression of ACKR3 appears sufficient to alter CXCR4-mediated tissue specific metastasis pattern in disseminated xenografts [26, 70]. DLBLC are known to invade the bone marrow and the CNS as well as other extranodal sites [38, 39]. The present results obtained with a xenograft model of the DLBCL cell line VAL show that ACKR3 expression is necessary for brain infiltration. In analogy with the poor prognosis of CNS infiltration by DLBCL in humans, mice injected with ACKR3^wt^ VAL cells showed a worse clinical score compared to animals injected with cells in which ACKR3 was genetically deleted. The observation is further supported by the *in vitro* observation that deletion of ACKR3 reduced the capability of the cells to transmigrate endothelial layers in response to CXCL12. Conditioning of the cells in mice lead to the upregulation of surface expression of ACKR3 which may additionally promote the aggressiveness of the cells *in vivo*.

Similarly, in the localized xenograft model ACKR3 expression correlated with invasion of draining lymph nodes, underlining the role of ACKR3 for cell mobility and aggressiveness of lymphoma. Several studies illustrate the ability of atypical receptors, such as ACKR3 and ACKR4, to create functional chemokine gradients [7, 13, 14]. Antagonizing scavenging activity of ACKR3 with small molecules or genetic deletion may interfere with the formation of local CXCL12 cues attenuating lymphoma cell dissemination. Taken together, the data unveil ACKR3 as a potential therapeutic target for DLBCL.

## MATERIALS AND METHODS

### Cell lines and cell culture

DLBCL cell lines were as previously described [71]. VAL and Karpas422 cells were cultured in RPMI-1640 medium complemented with 10% heat-inactivated FBS, 1% Penicillin/Streptomycin, 1% GlutaMAX. OCI-LY19, OCI-LY8, and the mouse pre-B cell line 300.19 were cultured in same medium supplemented with 1% non-essential aminoacids, 1% sodium Pyruvate, and 50μM β-mercaptoethanol. RIVA and SUDHL16 cells were cultured with IMDM, 10% heat-inactivated FBS, 1% Penicillin/Streptomycin. All media and supplements were purchased from Thermo Fisher Scientific. Human dermal lymphatic microvascular endothelial cells (hLECs) (Lonza) and primary murine lymphatic endothelial cells (pmLECs) where donated by Dr. Cornelia Halin Winter (ETH, Zurich). The hLECs were cultured in EBM-2 medium without VEGF-A (EGM-2 MV BulletKit Lonza); pmLECs where cultured in 40% DMEM (low glucose), 40% F12-Ham, 20% FBS (Thermo Fisher Scientific), 56 μl/ml heparin (Sigma), 10 μl/ml endothelial cell mitogen (Bio-Rad), cAMP (25 μg/ml), hydrocortisone (10 μg/ml) antibiotic/antimycotic solution (Sigma), and L-glutamin (2 mM, Sigma). Both, hLECs and pmLECs where cultured on dishes coated with collagen (10 μg/ml, PureCol, Advanced Biomatrix) and collagen plus fibronectin (MerkMillipore), respectively. All cells were cultured at 37°C with 5% CO_2_.

### Transmigration assay

The hLECs and pmLECs where cultured as monolayer on Transwell^®^ migration plates (5μm pores size, Corning). VAL cells (10^6^/ml) were resuspended in RPMI-1640 supplemented with 25mM HEPES, 1% FBS and 1% penicillin/streptomycin and added on top of endothelial cells. CXCL12 was diluted in medium supplemented with 10% FBS at the indicated concentrations and added to the lower compartments. Plates were incubated at 37°C for 6h. Migrated cells were recovered from the lower compartments and counted by flow cytometry.

### FACS analysis and cell sorting

Cells in FACS buffer (phosphate buffered saline (PBS) 2% FBS, 0.05% sodium azide) were stained for 15-30 min on ice with the appropriate antibodies. Cells were analyzed with a Becton & Dickinson (BD) LSRFortessa or FACSCanto I cytometers and FlowJo software. For sorting cells were stained with appropriate Abs, washed with FACS buffer and resuspended in PBS 2% FBS, 2 mM EDTA, filtered through a cell strainer and sorted with BD FACSAria IIIu.

### Antibodies

The following antibodies were used for FACS staining: anti-human ACKR3 clone 9C4 (IgG1) and anti-human CXCR4 clone 12G5 (IgG2a) as previously described [48] and counterstained with isotype specific goat antibodies (Southern Biotech). Anti-human ACKR3 clone 11G8-PE was purchased from R&D systems, anti-human CD19-PE-Cy7 clone SJ25C and anti-human CXCR4-PE clone 12G5 from BD Pharmingen and anti-mouse CD45-APC-Cy7 clone 30-F11 from BioLegend.

### Semi-quantitative and quantitative PCR

Total RNA was isolated with TRIzol Reagent (Ambion) and RNA concentration was determined. Reverse transcription of 2 μg of RNA was performed with the Superscript Vilo kit following the manufacturer’s instructions (Thermo Fisher Scientific). The following primers were used for semi-quantitative PCR and real time (RT)-PCR:

ACKR3 - 5’-ATGGATCTGCATCTCTTCGAC-3’ and 5’-GTAGCGGTCCACGCTCATGC-3’, β-Actin 5’-TCACCCACACTGTGCCCATCTACGA-3’ and 5’-CTAGAAGCATTTGCGGTGGACGATGG-3’. Amplification of cDNA obtained from *ex vivo* VAL cells was performed after CD19^+^ cells enrichment by magnetic separation with anti-human CD19 microbeads (MACS, Miltenyi Biotec). qPCR was performed using the PerfeCta SYBR Green FastMIX (ROX) (Quanta Bioscience) on MicroAmp Fast Optical 96-well Reaction plate (Applied Biosystem). Expression of the TATA-box binding protein (TBP1) was used as control: 5’GTTCTGGGAAAATGGTGTGCACAGGAGCCAAG3’ and 5’GCTGGAAAACCCAACTTCTGTACAACTCTAGC3’. Data were analyzed by means of ΔCt values and ACKR3 expression was calculated as mean difference of ACKR3 Ct values minus mean TBP1 ΔCt value, expressed as 2^-ΔCt^.

### Uptake assay of fluorescently labelled chemokines and anti-ACKR3 antibody

VAL cells were pre-incubated for 10 min at 37°C with the CXCR4 inhibitors AMD3100 (10 μM, Sigma-Aldrich) and the isothiourea 1a NIBR-1816 5 μM (kind gift from H.G. Zerwes, Novartis) [46], and the monoclonal antibodies 12G5 (anti CXCR4) 30 μg/ml, 9C4 (anti-ACKR3) 30 μg/ml. Cells were then incubated with medium alone or 50 nM chimeric chemokine or CXCL12 in the presence of absence of the inhibitors for 1 h at 37°C. Recombinant chemokines were produced and labelled as described [45]. Reactions were terminated by washing with PBS containing 2% FBS and cells subjected to a brief acidic wash [48]. For the antibody uptake assay, cells were incubated with α-human ACKR3 mAb 11G8-PE (1:100 diluted from commercial stock) at 4°C or at 37°C for 1 hour. Samples were briefly washed at low pH [48] and resuspended in FACS buffer analysis.

### ACKR3 gene knock out

CRISPR/Cas9 mediated gene editing of ACKR3 was performed by transfecting VAL cells with either of two guideRNA-Cas9 vectors (U6gRNA-Cas9-2A-RFP, Sigma) with target sites TCTTCGACTACTCAGAGCCAGG and AACAGCAGCGACTGCATCGTGG respectively in combination with a HDR-repair construct (in pUC57-Kan, Sigma) introducing CFP under the SFFV promotor. Briefly, two million VAL cells were nucleofected (Nucleofection Kit V, Lonza) with 1 µg guideRNA-Cas9 vector plus 4 µg NdeI-linearized HDR-repair plasmid (program X 01). Two days after transfection red fluorescent protein (RFP) positive cells were enriched by FACS-sorting and cultured for one week. Nucleofection was repeated with the second guideRNA-Cas9 vector plus the HDR-construct. After RFP-FACS enrichment, cells were cultured for one week followed by single cell sorting. Clones were tested for successful knock down of the *ACKR3* gene by genomic PCR using the following primers: 5’CAATGGTACCCCGTGGCTGAATTC3’ forward and 5’TTGCTCTAGAAAACCATAGGGCCCATC3’ reverse. One out of seven clones showed a single PCR product of 3311 nucleotides corresponding to the successful integration of the HDR-repair construct in both alleles, which was confirmed by sequencing.

### Mice and mouse models

All animal experiments were performed in accordance with the Swiss Federal Veterinary Office guidelines and authorized by the Animal Studies Committee of Cantonal Veterinary. Genetically immunosuppressed NOD.Cg-*Prkdc*^*scid*^ *IL2*^*rgtm1wjl*^*I* SzJ were purchased from Jackson Laboratory and bred in a specific-pathogen free facility. Mice, 6-8 weeks-old, were injected subcutaneously with 10^7^ VAL cells in PBS in order to create localized xenografts. Tumor growth was monitored daily, and when the tumors were palpable, they were measured with a digital caliper. Tumor volumes were calculated with the following formula: V= length × width^2^ × 0.5. Disseminated xenografts were produced through intravenous injection in the tail vein of 2×10^5^ Val cells in PBS. Mice were monitored daily and clinical scores were assessed as follow: Score 0 = no signs of discomfort, Score 1 = slight movement impairment, Score 2 = hind leg paralysis. Mice were latest sacrificed at score 2.

### Tumor processing and leukocyte isolation

Localized xenografts were surgically removed, and mechanically disintegrated between 150 μm Sefar Nitex filters. Cells were washed and cultured. In the disseminated xenograft model, mice were sacrificed and extensively perfused with PBS. Femurs, tibiae, lungs, spleen and brain were collected. Bone marrow cells were recovered from femurs and tibiae by gentle centrifugation and filtration. Lungs were minced with surgical scissors and digested with 0.05 mg/ml liberase (TL Research Grade) and 1 mg/ml DNase I (Sigma) in PBS for 1 h at 37°C. The digestion was terminated by the addition of medium and the cells separated from stroma by filtration. Brains were disintegrated between filters and incubated with 1 mg/ml DNase I and 125 μg/ml collagenase IV (Sigma) at 37°C for 30 min under agitation. For leukocyte isolation the cell suspension was filtered and mixed with 30% Percoll for leukocyte isolation by centrifugation was performed at 4°C and 353 × *g* for 20 min.

### Histology and immunohistochemistry

For histopathological analysis organs from mice from disseminated xenograft tumor models were harvested, rinsed with PBS and placed in 4% neutral buffered formalin (Thermo Scientific). Tissue specimens were embedded in paraffin. Paraffin sections (4 μm) were stained with hematoxylin and eosin (H&E). For immunohistochemical analysis, slides from all organs sampled (bone marrow, brain, lung, spleen, stomach, kidney, small and large intestines, lymph nodes and skin) were stained with a monoclonal mouse anti-human CD20 antibody (clone L26, prediluted, Ventana Medical Systems, Tucson, Ariz., USA). Following pretreatment according to the manufacturer's protocols, the slides were incubated at room temperature on an automated immunostainer (BenchMark XT, Ventana Medical Systems, Tucson, Ariz., USA). Antigen detection was performed using a commercial detection kit (UltraView Detection Kit; Ventana) with diaminobenzidin as the chromogen.

### Statistical analysis

All data are expressed as mean (±SD) and where analyzed with SigmaPlot. Statistical analyses were performed with two-tailed Student’s t-test or one-way ANOVA test. Two-sided p values less than 0.5 were considered significant.

## SUPPLEMENTARY MATERIALS FIGURES



## Abbreviations

ACKR3: atypical chemokine receptor 3
ALL: acute lymphoblastic leukemia
AML: acute myeloid leukemia
CFP: cyan fluorescent protein
CNS: central nervous system
DLBCL: diffuse large B cell lymphoma
GC: germinal center
GPCR: G-protein coupled receptor
hLEC: human dermal lymphatic microvascular endothelial cells
MALT: mucosa-associated lymphoid tissue
PBS: phosphate buffered saline
pmLEC: primary murine lymphatic endothelial cells
RFP: red fluorescent protein
TEM: transendothelial migration
