# Supplementary material for: ACKR3 expression on diffuse large B cell lymphoma is required for tumor spreading and tissue infiltration
Source: Oncotarget. 2017 Jun 29;8(49):85068–84. doi: 10.18632/oncotarget.18844 (PMC5689594; doi:10.18632/oncotarget.18844)
Supplement: Supplementary file 1 [file oncotarget-08-85068-s001.pdf]

ACKR3 expression on diffuse large B cell lymphoma is required for tumor spreading and tissue infiltration

SUPPLEMENTARY MATERIALS

A

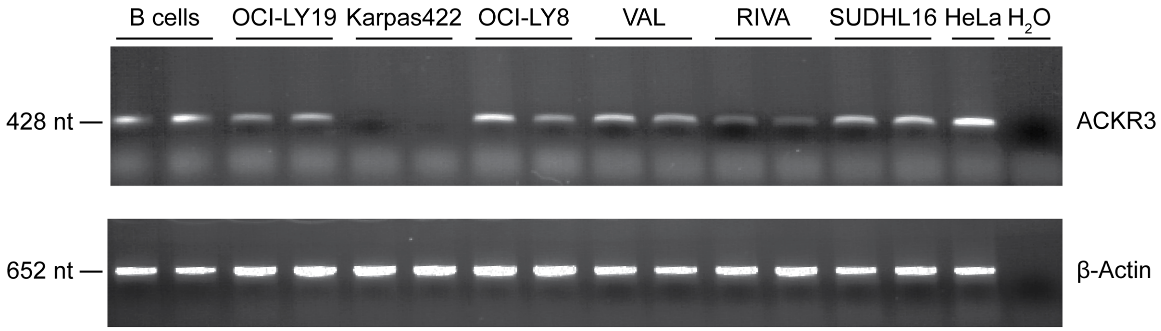

B

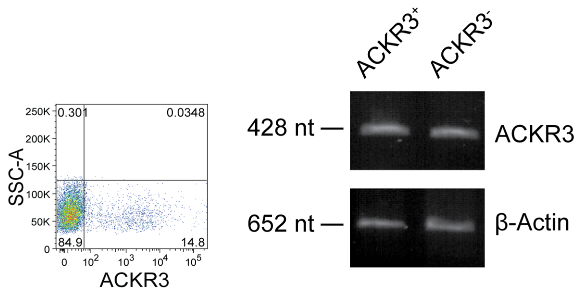

Supplementary Figure 1: (A) ACKR3 is expressed in selected GCB-DLBCL cell lines. Semi-quantitative PCR of ACKR3 transcripts is shown. ACKR3 is expressed on OCI-LY19, OCI-LY8, VAL, RIVA and SUDHL16 cell lines, but not on Karpas422 cells. Peripheral blood human B cells and HeLa cells were used as positive controls. β-Actin mRNA was measured as internal control. Representative data of one of at least three independent experiments. **(B)** Regulation of ACKR3 surface expression is independent from *ACKR3* gene transcription levels.ACKR3 surface expression was measured by flow cytometry using mAb 11G8. Cells were sorted for ACKR3 expression. $ACKR3^+$ and $ACKR3^-$ subpopulations of VAL cells (left panel) express similar mRNA levels (right panel) as revealed by semi quantitative PCR.

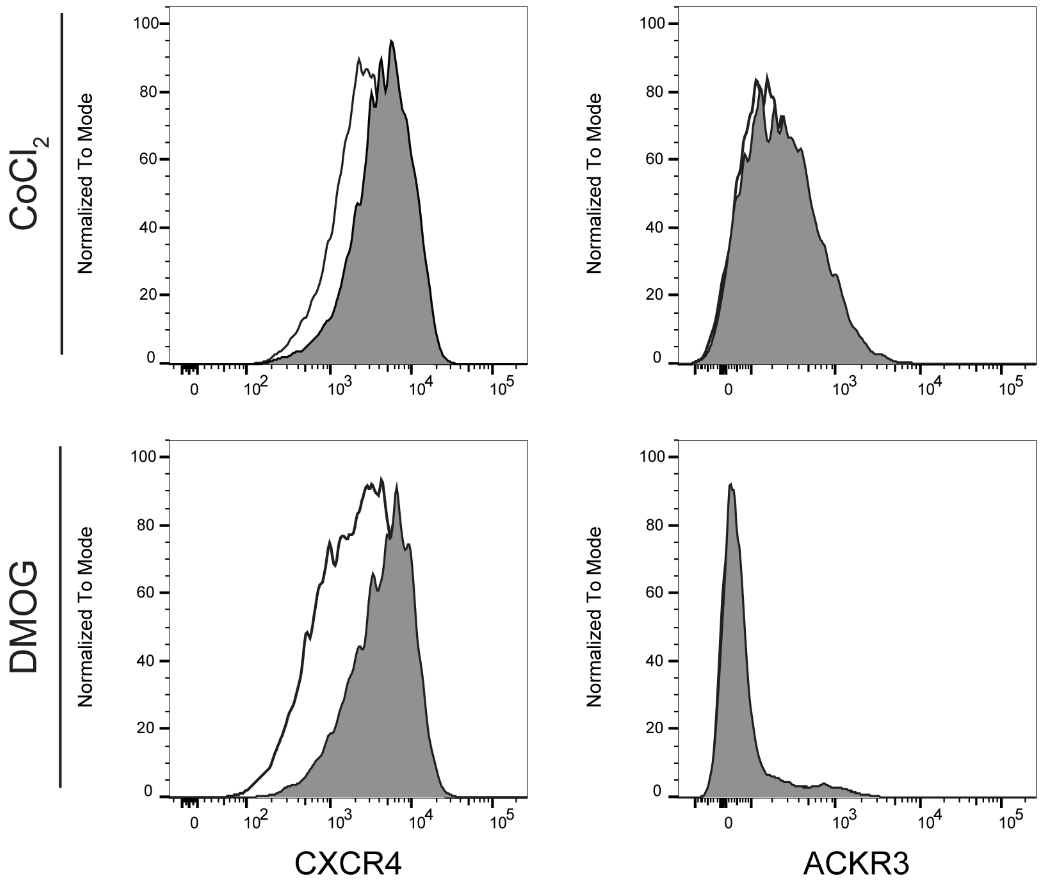

Supplementary Figure 2: CXCR4 (left) but not ACKR3 (right) is upregulated in response to hypoxia. VAL cells were cultured in the absence (open histograms) or in the presence of the hypoxia-miming compounds $CoCl_2$ (100 µM) or DMOG (500 µM) (grey histograms). Surface expression of ACKR3 (mAb 9C4) and CXCR4 (mAB 12G5) were evaluated by flow cytometry. Representative plots of three independent experiments.

A

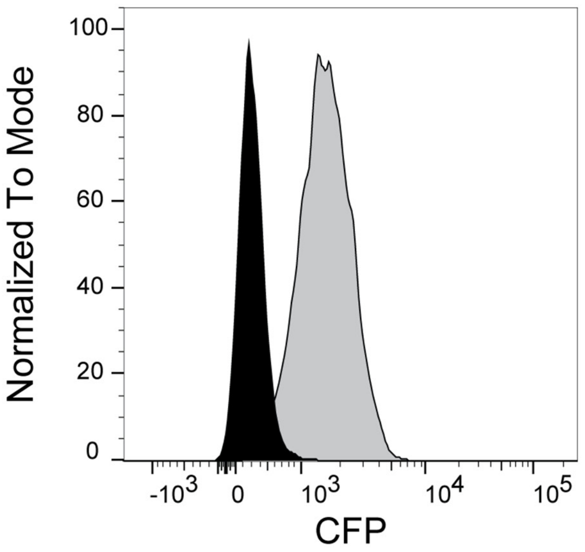

B

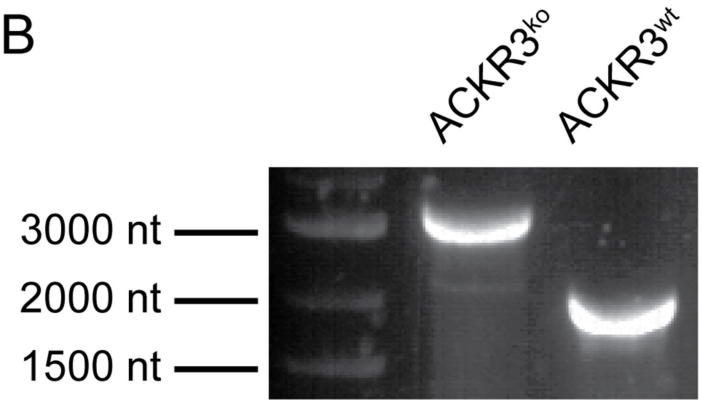

Supplementary Figure 3: (A) Expression of CFP in ACKR3^{ko} cells (gray) analyzed by flow cytometry. ACKR3^{wt} cells (black) were used as control. **(B)** PCR of genomic DNA from ACKR3^{ko} and ACKR3^{wt} cells. PCR analysis of ACKR3 genomic DNA in wild type and CRISPR/Cas9 treated VAL cells. Primers were designed in order to cover the 5' genomic DNA region up to the 3' outside regions flanking of *ACKR3* gene, so that the predicted lengths of amplified fragments are 3312 nt in case of insertion of the HDR construct (ACKR3^{ko} cells) and 2128 nt in the original.

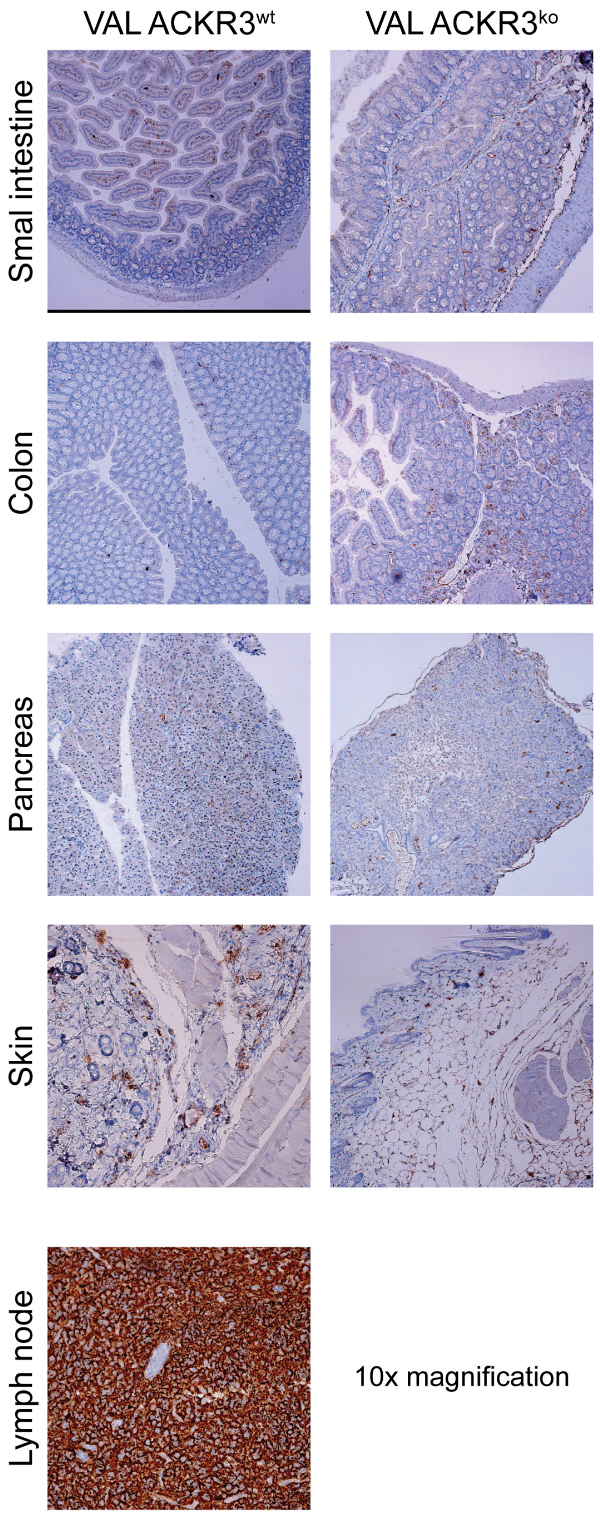

Supplementary Figure 4: Immunohistochemistry of tissue invasion by VAL ACKR3ko and VAL ACKR3wt cells. NOD/SCID/common γ-chainko mice were injected with 2x10^5 cells. Organs were removed after four weeks, formalin-fixed, paraffin-embedded and sections stained for CD20 (brown) expressing human VAL cells and counterstained with hematoxylin (blue).
